# Chronic Misuse of Paracetamol in OCD Without Hepatic Injury: A Case Report and Literature Review

**DOI:** 10.1192/bjo.2022.364

**Published:** 2022-06-20

**Authors:** Ashma Mohamed, Alexa Wacker, Martin Schmidt

**Affiliations:** 1 Surrey and Borders Partnership NHS Foundation Trust, Surrey, United Kingdom; 2 Ross University School of Medicine, St Michael, Barbados; 3 Head of School of Psychiatry, Surrey, United Kingdom

## Abstract

**Aims:**

Paracetamol is a commonly used antipyretic and analgesic over the counter medication. In acute or chronic overuse it is associated with dose-dependent hepatic injury. There is a narrow therapeutic margin and that consistent use of as little as 7.5 g/day may be hazardous. Unintentional overdose with paracetamol is the most common cause of acute liver failure in the United Kingdom Here we present an unusual case of a 60-year-old lady with a reported chronic history of self-medicating with an above daily recommended dose of paracetamol without evidence of hepatic injury.

**Methods:**

A 60-year-old Caucasian lady known to psychiatric services for 20 years with Recurrent Depressive disorder, Obsessive Compulsive Disorder (OCD), Dependent Personality Disorder with Borderline personality traits. She reported consuming 32 tablets of paracetamol (16gm per day) every day for the past 11 years. She experienced obsessions of fear that if she did not take a particular number of paracetamols in a day then her friends will come to harm and her anxiety was relieved by the compulsion of consuming supratherapeutic doses of paracetamol. There was no evidence of misuse of any other medications other than paracetamol. Her blood investigations revealed liver function tests within normal limits and ultrasound of the liver was unremarkable.

**Results:**

A literature search of “paracetamol or acetaminophen” and “no liver or hepatic” and “damage or injury” found only one case report. The case reported that studies of paracetamol metabolism were performed in a 58-year-old female with rheumatoid arthritis who had consumed 15–20 g paracetamol daily for 5 years without developing liver damage and data were compared with results in seven normal volunteers. The report concluded that a combination of slow paracetamol absorption, enhanced detoxication of paracetamol (by sulphation) and reduced metabolism to potentially cytotoxic metabolites may have reduced the risk of liver damage in this patient.

**Conclusion:**

In OCD, misusing medications can be an uncommon presentation of compulsive acts to relieve anxiety. The diagnostic dilemma of factitious illness is probable, however supratherapeutic use of paracetamol without physical harm is rare but possible.

